# A Novel Cost-Efficient Framework for Critical Heartbeat Task Scheduling Using the Internet of Medical Things in a Fog Cloud System

**DOI:** 10.3390/s20020441

**Published:** 2020-01-13

**Authors:** Qurat-ul-ain Mastoi, Teh Ying Wah, Ram Gopal Raj, Abdullah Lakhan

**Affiliations:** 1 Faculty of Computer Science and Information Technology, University of Malaya, Kuala lumpur 50603, Malaysia; 2 School of Computer Science and Engineering, Southeast University, Nanjing 211189, China; abdullah@seu.edu.cn

**Keywords:** task scheduling, cost, ECG sensors, heartbeat, health care based fog cloud system (HCBFS), health care awareness cost-efficient task scheduling (HCCETS) algorithm, task prioritization

## Abstract

Recently, there has been a cloud-based Internet of Medical Things (IoMT) solution offering different healthcare services to wearable sensor devices for patients. These services are global, and can be invoked anywhere at any place. Especially, electrocardiogram (ECG) sensors, such as Lead I and Lead II, demands continuous cloud services for real-time execution. However, these services are paid and need a lower cost-efficient process for the users. In this paper, this study considered critical heartbeat cost-efficient task scheduling problems for healthcare applications in the fog cloud system. The objective was to offer omnipresent cloud services to the generated data with minimum cost. This study proposed a novel health care based fog cloud system (HCBFS) to collect, analyze, and determine the process of critical tasks of the heartbeat medical application for the purpose of minimizing the total cost. This study devised a health care awareness cost-efficient task scheduling (HCCETS) algorithm framework, which not only schedule all tasks with minimum cost, but also executes them on their deadlines. Performance evaluation shows that the proposed task scheduling algorithm framework outperformed the existing algorithm methods in terms of cost.

## 1. Introduction

Previous research has shown that the ratio of mortality due to heart diseases increase day by day. According to the American Heart Association and the World Health Organization, about 735,000 Americans suffer from heart disease [1]. It reveals that about 230 million patients have cardiovascular disease (CVD), with 3 million deaths annually [2]. To spot heart irregularities, electrocardiography (ECG) signals are the primary source of evaluation that is widely used by medical specialists arround the world [3]. However, due to the sporadic nature of ECG signals, it is necessary to monitor patients continuously to have for accurate analysis of the heart problems [4]. Recently, advancements in Internet of Things (IoT) based medical sensors have grown progressively [5,6,7,8,9,10,11,12,13,14,15]; especially in heartbeat sensors that generate real-time delay-sensitive data that require immediate action for the results [16,17]. Generally, these sensors are integrated with limited constraint devices. Thus, fog computing is a promising and delay-efficient paradigm, where computing and capability are offered at the edge of IoT network [4,18,19]. It is noticed that each heartbeat-based medical application is composed of critical tasks and less delay-sensitive tasks. Therefore, a fog paradigm is efficient for the sensor data in healthcare medical applications; however, different fog servers have different costs for data execution [20,21,22,23]. All medical services are paid in the fog cloud server networks, therefore cost-efficient task scheduling for medical IoT applications is a challenging task.

In [24], the author proposed the reservoir computing-based cyclic echo state-network for ventricular, (critical) heartbeat classification. The proposed algorithm was specially designed for implementation in medical wearable wireless gadgets as it is fast, with less power consumption, and can be easily adaptable to small hardware devices. The main purpose was to propose a cost efficient-based approach for ventricular heartbeat detection in real-time scenarios. In the current paper, the authors extend their previous work, [24], to provide a cost-efficient solution for high priority (critical) heartbeat task scheduling. A novel framework named the HCCETS framework was proposed to minimize the cost of heart beat-based healthcare applications during task scheduling. Each heartbeat medical application is composed of autonomous fine-grained tasks. There are different types of tasks involved in the application, for instance, critical tasks that lead to severe heart disease or sudden cardiac death (those that required immediate action for the processing) and non-critical tasks (that has long deadlines and is to be processed anytime). Every task has an original workload for processing under a given deadline; for the considered problem, the different types of fog servers were taken into account for processing the requested tasks. Each fog server is distinct by its speed, capacity, and cost.

**Objective:** The Internet of Medical Things (IoMT) application is a particular type of application that runs different services via sensors. For instance, heartbeat control and blood pressure tasks exploit various services to facilitated patients efficiently, whereas, the IoMT application consists of critical and non-critical tasks. Critical tasks require immediate services to run the operation and patients in dangerous heartbeat situations. However, these services are not free and are offered by the hospital to patients. Therefore, in this paper, the author only focused on a cost-efficient task scheduling problem to schedule all critical heartbeat tasks on servers in order to reduce maximum costs to meet the right situational requirements.

In the literature, numerous studies have addressed the issue of task scheduling for healthcare applications in the cloud system. For example, [25,26,27] investigated task scheduling in the fog cloud system for the medical system. The objectives were either to minimize the devices’ energy or optimize delay during task scheduling in the fog cloud system. Moreover, there are many challenges to be addressed on the task scheduling problem in the fog cloud system. These questions are as follows: (i) How to prioritize critical tasks of an application; (ii) How to find an optimal time-slot in the fog cloud in order to meet the QoS of tasks; (iii) How to schedule tasks onto different fog cloud networks in order to minimize the average cost of an application under its QoS requirements.

This paper makes the following main contributions based on the questions mentioned earlier.

- In other to solve the cost-efficient task scheduling problem for critical heartbeat conditions, we formulated this problem as a scheduling problem. Generally, the task scheduling problem requires multiple steps to address the cost-efficient assignment of tasks onto heterogeneous resources. To have this problem solved, we proposed a health care awareness cost-efficient task scheduling (HCCETS) algorithmic framework that is composed of the following phases: a task prioritizing phase, a resource searching phase, and a task scheduling phase.
- Task prioritizing phase: Generally, the ECG signal is the primary source for the monitoring of electric cardiac activity of the heart. Every ECG cycle provides various types of information regarding the patient; for instance, if the patient has an arrhythmic heartbeat, a myocardial infarction, coronary artery disease and so on. In this phase, the author prioritized the critical tasks of heartbeat for an immediate process for execution, as these tasks were considered as an essential task. On the other hand, the delay-tolerant tasks, likewise patient report tasks, do not require a necessary process for execution. To handle the priority of urgent and late tasks, the author proposes a new task sequence rule method, which is not only used to satisfy the execution requirement but also to minimize the average cost of the application.
- Resource search phase: The author considered different fog servers with their unique characterizations. Every fog server offers on-demand-based cloud services, to run the IoT heartbeat-based healthcare application. The characterization of each server is a set of a vector, such as computing capability, storage, and price. Therefore, costs and deadlines are critical factors when the system chooses a fog server to run the tasks of the IoT application. To cope with this situation, the author proposes an efficient resource algorithm that is capable of utilizing the appropriate resource for each task to reduce the cost.
- Task scheduling phase: Task scheduling is a critical phase that allocates all dangerous heartbeat tasks into heterogeneous resources to assure work done at minimal cost accordingly. The author proposes a new cost-efficient task scheduling algorithm, which allocates all critical heartbeat tasks into appropriate resources until no tasks are left behind. To evaluate the effectiveness and efficiency of the proposed cost-efficient algorithm, the author compared it to the existing task scheduling algorithm when it was run over the heartbeat dataset of IoT applications.
- The author proposes the health care based fog system (HCBFS) that processes all requested heartbeat critical tasks to fog cloud networks.

The rest of the paper is organized as follows. Section 2 elaborates related works and Section 3 explains the problem description and formalizes the problem under study. A heuristic solution is proposed for the considered problem in Section 4, which describes the proposed algorithm and sequences. Section 5 evaluates the simulation and Section 6 is about the conclusion.

## 2. Related Work

Recently, the cloud-based Internet of Things (IoT) heartbeat medical applications have grown progressively due to global services to heart patients. Generally, different healthcare sensors generate data for heart patients and offload these data to the hospital fog server for further processing. Therefore, the scheduling of these data with different operations is a critical question. Numerous task scheduling problems for healthcare applications in the cloud system have been investigated in the literature. For the purpose of minimizing total delay, the author has focused on the studies related to offload healthcare tasks. During the last era of technology, highly intensive research activities took place in the area of IoMT. Many studies have presented their works, based on portable health care devices, for instance, [3,28] proposed computational frameworks for healthcare monitoring systems in mobile environments [20], and presented fog-computing based heartbeat detection for arrhythmia classifications. Patient-centric heart monitoring systems [28] using fog computing were proposed, the system established a connection between patient and medical specialists to perform the efficient operation of detecting abnormality in the heartbeat. Generally, state-of-the-art approaches have mainly focused on heart arrhythmia, and heart disease prediction from the non-invasive attributes of the morphological structure of the beat. However, the study deals with minimizing the delay sensitive task, and scheduling issues in critical heartbeat detection.

Whereas these studies [29,30,31,32] have focused on delay optimal task scheduling or task assignment problems in the fog cloud environment for heart-beat healthcare applications, the goal of the aforementioned is to minimize the total cost of and delay of each application during processing to the cloud system.

Furthermore, the task assignment and task offloading problems related to the healthcare applications are formulated in these studies [16,18,31,33]. The prior studies have focused on how to offload computation tasks to the cloud system in order to improve application performance on the user’s devices and measure the delay optimal results of healthcare data without any risk. The delay and cost-optimal task scheduling of heartbeat healthcare applications into cloud networks were investigated in [34,35,36,37]. The studies accepted the input of data from real-time sensors and provided the application tasks for the actions. These actions are performed by different clouds with respect to application requirements and their constraints.

To the best of this author’s information, cost efficient task scheduling for healthcare applications in fog cloud networks has not been investigated yet. The author considered both types of tasks, critical and non-critical, during scheduling in order to minimize the average cost of the application. Generally, the paper enhances user application and minimizes the cost in order to give vast benefit to the customers.

## 3. Problem Description

**Figure 1:**
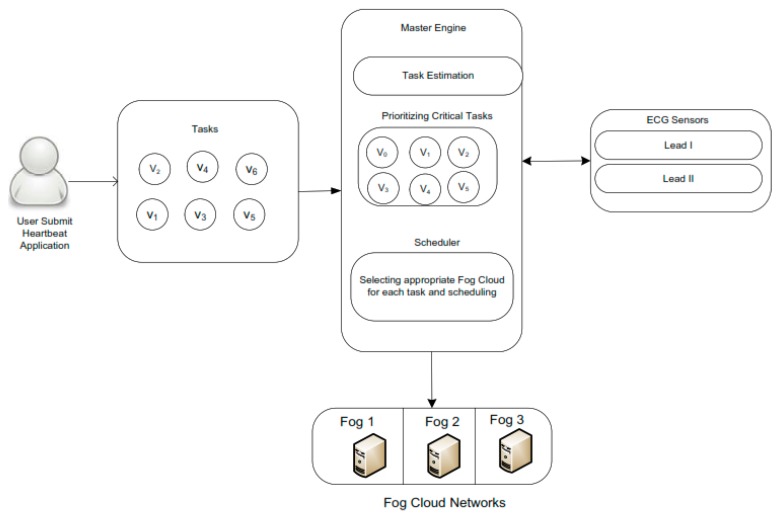
Healthcare Based Fog System.

The author has formulated a cost-efficient task scheduling algorithm for healthcare based heartbeat medical application tasks in the fog cloud networks. The heterogeneous fog cloud networks were used, with different costs and resource specifications for this purpose. The objective of the scheduling problem is to minimize the total cost of each task during the process in the fog cloud network. The propose healthcare based fog system (HCBFS) is a combination of different components. These components are master node, prioritizing critical tasks, scheduler, and ECG sensors, as depicted in Figure 1. The master node accepts requested tasks in the system, and estimates the task execution time of each task. Time-critical tasks are get higher in the prioritizing critical tasks component. Real-time data related to the tasks are continuously generated by the ECG sensors, such as Lead I and Lead II.

### 3.1. System Model

The author has formulated scheduling problems with different fog cloud servers with autonomous tasks. Each task works independently and it has its own data and specification. The arrival of tasks to the system is followed by the Poisson process. Each fog server offers exponential service to the offloaded tasks.

### 3.2. Application and Resource Model

The author has assumed that the healthcare application was composed of different tasks which are depicted as $\left\{v_{1},v_{2},v_{3},\ldots ..v_{n}\right\}$. Every task has its own workload, W*_i_* (*i* = 1, …, N), and latency deadline, $d_{i}$. The healthcare based fog system (HCBFS) is made up of heterogeneous fog cloud servers that are denoted by $\left\{V_{1},V_{2},V_{3},\ldots .V_{M}\right\}$. However, the fog servers are heterogeneous, therefore, each fog server has different computation speed and cost, which are depicted as $\zeta_{j}$ (j = 1, …, M) and $p_{j}$ respectively. To minimize the cost of offloaded tasks, the author assigned each task to the low-cost fog servers that satisfy the deadline $d_{i}$ constraint of a task. The author denoted the binary variable $x_{ij\;}\in \left\{0,1\right\}$ to show only if the task $\upsilon_{i}$ is assigned to the fog server $V_{j}$. The cost of each task $\upsilon_{i}$ on fog server is determined by the $c_{j}$, as well as execution time $T_{i}^{e}$, i.e., $T_{i}^{e}=\sum_{j=1}^{M}x_{i,j}\times \frac{W_{i}}{\zeta_{j}}$. Mathematic notations are listed in Table 1.

### 3.3. Mathematical Model

The considered cost optimization task scheduling problem is mathematically formulated as follows:

(1) $$minZ=\sum_{i=1}^{N}\ldots .\sum_{j=1}^{M}x_{i,j}\times c_{j}\;\times T_{k}^{e}$$

(2) $$T_{j,0}=0$$

(3) $$T_{j,k}=T_{j,k-1}+\sum_{k=1}^{N}x_{k,j}T_{k}^{e}$$

(4) $$T_{i}^{e}=\sum_{j=1}^{M}\cdot x_{i,j}\times \frac{W_{i}}{\zeta_{j}}$$

(5) $$F_{i}=\sum_{j=1}^{M}\cdot T_{j,k}x_{i,j}$$

(6) $$F_{i}\leq d_{i}$$

(7) $$\sum_{i=1}^{N}\cdot x_{i,j}=1$$

(8) $$\sum_{j=1}^{M}\cdot x_{i,j}=1$$

(9) $$x_{i,j}\in \left\{0,1\right\}$$

Equation (1) shows the objective function. Equation (2) shows the initial finish time of fog server, j, for task scheduling. Equation (3) shows the setup of a task on a fog server. Equation (4) determines the execution time of a task on all fog servers. Equations (5) and (6) show task finish time, which must be less than the given deadline. Equations (7)–(9) denote an assignment of a task only on fog servers and vice versa, with binary variable.

## 4. Proposed HCCETS Framework

**Figure 2:**
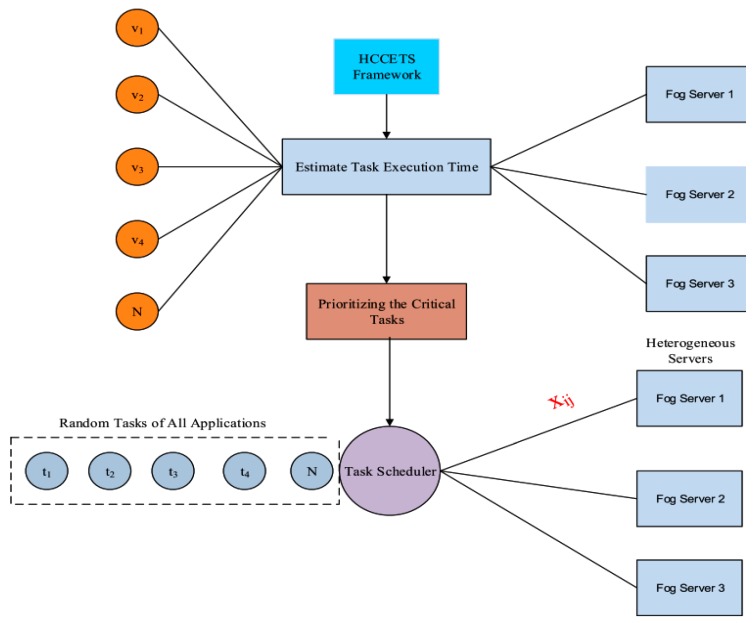
Diagram of HCCETS Framework.

The author formulated the task scheduling problem, which is a well known NP-hard problem. The author could not solve the task scheduling problem with one algorithm, because it required a multiple-step for the solution. For the considered problem, the author proposed a health care awareness cost-efficient task scheduling (HCCETS) framework made up of different components, as shown in Figure 2. The author solved the considered problem into a separate process, likewise task sequencing, initial task scheduling, critical task reshuffling, and cost efficient rescheduling. These components are illustrated in Algorithm 1. Where $Q_{\upsilon }$ *i*s the queue of different tasks is in the system, $Q_{d}$ is the list of task deadlines.
**Algorithm 1:** HCCETS Framework    **Input**: $Q_{\upsilon }$**;**
$Q_{d}$**;**
*{*$V_{j,1}$*, …,*
$V_{j,m}$}1    **begin**2      Z ← 0;3      **Call Task Sequencing**;4      **foreach** ($\upsilon_{i}$ ∈ $Q_{\upsilon }$) **do**5          Z*_i_* ← **Call Initial Task Scheduling**;6          Z ← Z + Z*_i_*;7          **Call Critical Task Reshuffling**;8          **Call Cost-Efficient Rescheduling**;9          Z* ← Z + Z*_i_*;10      **return** Z*; 

### 4.1. Task Sequencing

There are two types of tasks in the healthcare application, for example, time-critical tasks and less time-sensitive tasks. The critical tasks (e.g., emergency range of heartbeat or related operations) would get high priority. The normal reports related to the tasks get lower priority. Therefore, the author prioritized each task based on its requirements, such as deadline and workload. However, because of the heterogeneity of the fog serves, $T_{i}^{e}$*,* we devised the finish time of a task anticipatory of scheduling. The average execution time $\bar{T_{i}^{e}}=\;\frac{\sum_{j=1}^{M}W_{i}}{\sum_{j=1}^{M}\zeta_{j}}$ is estimated as

(10) $$T_{i}^{slack}=d_{i}-\;\bar{F_{i}}$$

(11) $$F_{i}=\;\sum^{\;}\bar{T_{i}^{e}}\;\frac{\sum_{j=1}^{M}W_{i}}{\sum_{j=1}^{M}\zeta_{j}}$$

The author prioritized all tasks by the following proposed sequence.

(1) Earliest Deadline First (EDF): The author sorted the set of tasks based on their deadline. The small deadline task is sorted first. If the deadline is the same, the task with the smaller size is ranked with a higher priority.
(2) Smallest Slack Time First (SSF): The tasks are sort according to the task slack time. The task which has smallest slack time is scheduled first. If the slack time is the same as any tasks, the smallest total workload will be arranged first.
(3) Smallest Workload First (SWF): The task is sequenced based on the size of the task, the smallest workload task is arranged first.

The generated sequences are as followed.

- EDF-based task sequencing: $\left\{v_{1},v_{2},v_{4},v_{3},v_{6},v_{5}\right\}$
- SSF-based task sequencing: $\left\{v_{1},v_{3},v_{5},v_{2},v_{6},v_{4}\right\}$
- SWF-based task sequencing: $\left\{v_{2},v_{4},v_{6},v_{5},v_{1},v_{3}\right\}$

The authors tried all sequences during initial task scheduling until the submitted tasks were satisfied with their requirements. 

### 4.2. Task Scheduling

The task scheduling phase schedules each offloaded task to the heterogeneous cloud based on their costs under the QoS requirement. The cost of each task, when it is to be assigned on any fog server, is denoted by, i.e., $c_{j}$ and the task execution time $T_{i}^{e}$. The cost of each task on fog server j as

(12) $$c_{ij}=\frac{\zeta_{j}}{P_{j}}$$

$c_{ij}$ is the unit cost of each fog cloud server when any task is assigned. All fog servers are sorted according to the $c_{ij}$ with the descending order and the available time $T_{j,0}$ of each fog server $V_{j}$ is initialized to 0. If $T_{j,i-1}+T_{i}^{e}<d_{i}$, then the fog server $V_{j}$, $\upsilon_{i}$ is identified, and the available time $T_{j,i}$ is dynamically updated. The details of the task scheduling on all fog cloud servers for all tasks is described in Algorithm 2.

1. In line 2, all fog servers are sorted by calculating $c_{ij}$ with the descending order and put into $Q_{\upsilon m}$ in which the fog servers are iteratively traversed.
2. In line 3, initially, all fog servers are null.
3. The available time $T_{j,0}$ of each fog server in the $Q_{\upsilon m}$ is initialized to 0.
4. Line 7 to 11, if the available time of the fog server $V_{j}$ plus the execution time of $\upsilon_{i}$ is less than the deadline $d_{i}$, $v_{i}$ is assigned to the fog server $V_{j}$, and the new available time $T_{j,i}$ of $V_{j}$ is dynamically updated.

**Figure 3:**
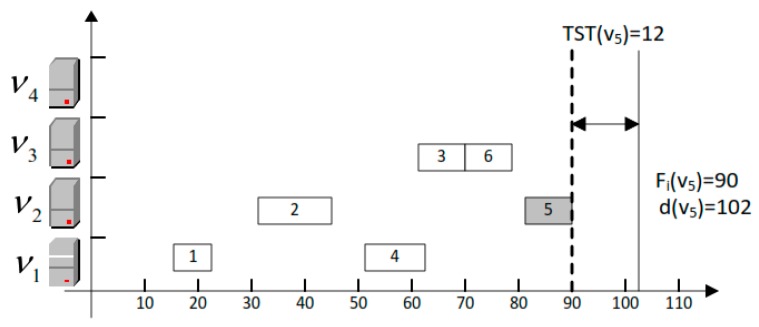
The task *v*_5_ with *TST (v*_5_ *= 12)* sequence adjustment.

**Figure 4:**
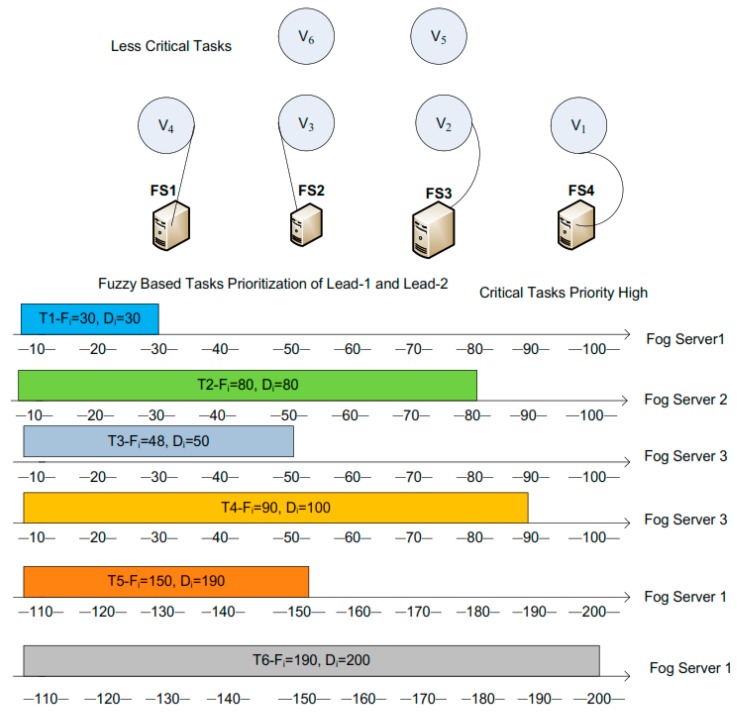
Random and cost-efficient fog servers searching.

The fog servers are sorted in Algorithm 2, the fog servers are swapped at least *M* × *log*(*M*) times. Besides, the traverse of the sorted fog servers consumes *M* times, therefore, the time complexity of Algorithm 2 is *O*(*M* × *log*(*M*)). The most cost-efficient unoccupied fog server is acquired in $Q_{\upsilon m}$ while satisfying the deadline $d_{i}$ of the task $\upsilon_{i}$. The task scheduling rule is compared to obtain the fog server with minimum cost for the task $\upsilon_{i}$. This mechanism guarantees that the finish time $F_{i}$ of task $\upsilon_{i}$ is equal with or smaller than the deadline $d_{i}$. For tasks with smaller $F_{i}$ than $d_{i}$, in most circumstances, generally, the result of the TST, $TST\left(\upsilon_{i}\right)$, is the difference between $F_{i}$ and $d_{i}$. Figure 3 illustrates an example of the task $\upsilon_{5}$ with $TST\left(\upsilon_{5}\right)=12$. To fully exploit TST, next task starts to execute as the first finish method is proposed to reclaim the TST. Supposedly, the study has many tasks which are indifferent workloads and deadlines. Every task has a different slack time. So the study has proposed an algorithm like that, when a task is finished in execution then server starts to execute the next task. The selection of a right fog server to execute tasks while minimizing cost optimization is very critical. If we do not find the right fog server for an assigned task then it would consume more cost and resources. Hence, the study needs to schedule all tasks on a variety of fog servers in a cost-efficient way. Figure 4 shows the difference between random fog server searching and cost-efficient fog server searching. The author could see the difference between both methods—which is more cost-efficient and meets the user-defined deadline. In this example of the figure, the study has six tasks that have different workloads ready for execution over four fog servers. All the fog servers are heterogeneous and have different processing capacities. So these six tasks are going to be scheduled over these four fog servers with the lowest cost. First, in the random fog server searching method, the author could see the six tasks take all four fog servers for their execution. In addition, some tasks have finish times exceeding the defined deadline and some resources are wasted. But in the second cost efficient fog server searching method, the author could see it took just three fog servers to execute all the tasks and all the tasks were finished within their deadlines while minimizing unit cost of fog servers. This means that the right fog server for scheduling a task is very helpful in reducing computation resource costs.
**Algorithm 2:** Initial Task Scheduling    **Input**: *v_i_*:task to schedule1  PList[*v_i_* ∈ N, j ∈ M];2  **begin**3      *Q_vm_* ←Sort the fog serves by the cij with the descending order;4      *V* ← NULL;5      **foreach**
*V_j_* ∈ *Q_vm_*
**do**6          *T*_j,0_ ← 0;7      **foreach**
*V_j_* ∈ *Q_vm_*
**do**8          Calculate the *T_i_^e^* of *V_j_* by the Equation (4);9          **if**
*T_j,i -1_* + $T_{i}^{e}$ < *d_i_*
**then**10          Calculate the *T_j,i_* of *V_j_* by the Equation (3); 11          *Z*
*←*
*V_j_;*12          break;13      Calculate cost of Z by the Equation (1);14      *PList[v_i_ ∈ N, j ∈ M] ← Z;*15      **return**
*Z*, *V*;

### 4.3. Critical and Non-Critical Tasks

*PList[v_i_* *∈* *N, j* *∈* *M]* is the preference list, in which this study stores the cost of the model for all tasks on each cloud during initial scheduling. As this study suggests, some normal tasks can be changed into critical tasks. For instance, if the normal heartbeat task range increases from low range to a higher range, the patient heart health would become critical. Algorithm 3 handles this situation: if the task changes their initial running status (e.g., normal task to critical), Algorithm 3 implicitly changes their priority and the new critical task would get high priority. The study swapped the time-slot of each task during the run-time of the task in order to handle any sensitive condition.
**Algorithm 3:** Critical Task Reshuffling    **Input**: *Z, PList[v_i_**∈** N, j ∈ M];*1  **begin**2      **foreach**
*(v_i_ as N)*
**do**3          **foreach**
*(j = 1 as M)*
**do**4                 **if**
*(*$T_{i}^{e}$*.v_i_ >*
$T_{i}^{e}$*.N)*
**then**5                     Swap: *v_1_*
*←*
*v_2_;*6                     Calculate the *T_j,i_* of *V_j_* by the Equation (3);7                         *V*
*←*
*V_j_*;8                          Z*
*←*
*V*; break;9                 PList[v_i_ ∈ N, j ∈ M] ← Z*;10               **return**
Z*, PList[v_i_
*∈ N, j*
*∈ M]*; 

### 4.4. Cost-Efficient Rescheduling

The study rescheduled all tasks on different fog servers with respect to their deadlines and cost, as shown in Algorithm 4. The output of Algorithm 4 is shown in Figure 3. All scheduled tasks are rescheduled in a way that all critical tasks with respective deadlines and cost are to be obtained with high priority, and are to be scheduled first. The less delay-sensitive tasks, with respect to their deadlines and cost, are scheduled later.
**Algorithm 4:** Cost-Efficient Rescheduling    **Input**: *Z, PList[v_i_ ∈ N, j ∈ M];*1  **begin**2      **foreach**
*(v_i_ as N)*
**do**3          Calculate the $T_{i}^{e}$ of *V_j_* by the Equation (4);4            **if**
*T_j,i -1_* + $T_{i}^{e}$
*< d_i_*
**then**5                Calculate the *T_j,i_* of *V_j_* by the Equation (3);6                *V*
*←*
*V_j_*;7                break;8            Calculate cost of Z by the Equation (1);9            PList[v_i_ ∈ N, j ∈ M] ← Z*;10          **return**
Z*;


### 4.5. Time Complexity

The proposed algorithm exploits *O*(*n*|*log*|*m*) time complexity, where *n* is the number of iterations for all tasks when they are assigning to the numbers of *m* fog cloud servers.

## 5. Performance Evaluation

### 5.1. Practical Implementation of (HCBFS) 

**Figure 5:**
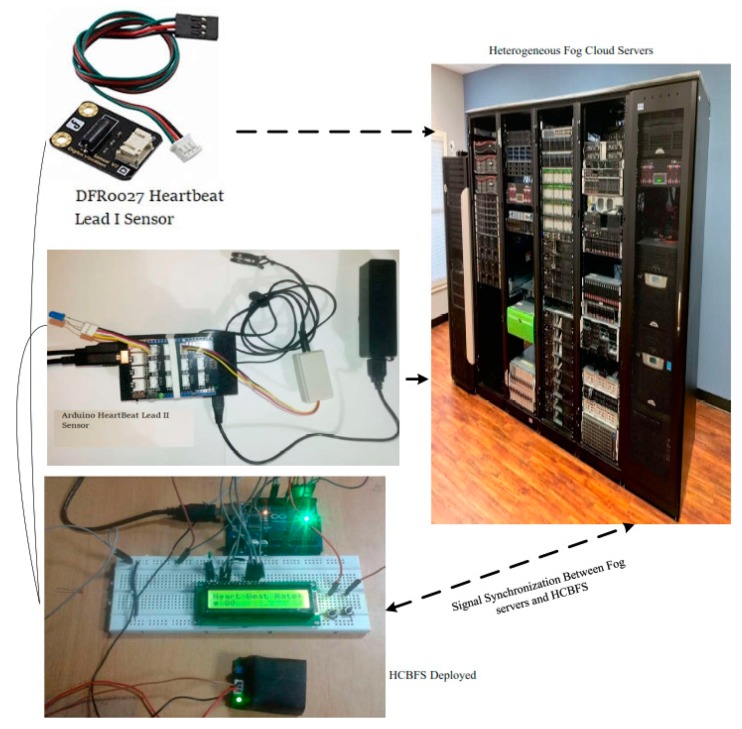
Health care based fog cloud system.

This study developed the health-care based fog cloud system using different sensors such as Arduino and DFR heartbeat sensors. These sensors are connected to the fog system via the HCBSF system which is developed in the JAVA language, as shown in Figure 5. Both sensors generate real-time data for different tasks. Some of them are critical tasks; for instance, patient has observed abnormal rhythm of the heart. These types of critical tasks are required to perform their actions into the fog cloud based on the provided information by the sensors. These practical setups are implemented at the University of Malaya advance robotics lab (Table 2). Initially, this study generated the data from sensors which were synchronously exchanged between fog servers and HCBFS, while performing healthcare application tasks. This study developed a healthcare mobile application, based on JAVA and perform its actions based on sensor data. Furthermore, the same experiments were conducted on three public datasets, namely AHA [38], MIT-BIH-SVDM, and MIT-BIH-AR [39]. 

### 5.2. Resources Specifications

This study considered the heterogeneous fog servers refer to systems that use more than one kind of processor or core. These systems gain performance or cost efficiency not just by adding the same type of processors, but by adding dissimilar coprocessors, usually incorporating specialized processing capabilities based on on-demand services. This study shows the characterization of each cloud fog cloud server in Table 3.

### 5.3. Heartbeat Datasets

This study used three different public benchmark datasets for the efficiency and effectiveness of the proposed algorithm, namely, the MIT-BIH Supraventricular Arrhythmia database (MIT-BIH-SVDM), the MIT-BIH-Arrhythmia database (MIT-BIH-AR) [39] and the American Heart Association database (AHA) [1]. The overall description of abovementioned datasets is defined in Table 4. MIT-BIH-SVDM includes 78 half-hour ECG recordings; the AHA dataset represents information that is directly provided by nearly 6300 hospitals and more than 400 health care systems, whereas the MIT-BIH-AR dataset contains 44 ECG subjects with five major classes of arrhythmia, namely, non-ectopic beat (N), supraventricular ectopic beat (S), ventricular ectopic beat (V), fusion beat (F), and unclassified and paced beat (Q). According to ANSI/AAMI standards, four recordings (102, 104, 107, and 217) containing paced beats; due to that, the signals did not retain sufficient signal quality for signal processing. This study evaluated the efficiency and effectiveness of existing cost-efficient task scheduling algorithms and proposed an algorithm based on the given heart beat datasets. For the existing algorithms, annotated as Baseline1 and Baseline 2, [40,41] have conducted their experimental results by exploiting datasets, as discussed above. However, it is convenient to evaluate the performance of all algorithms based on similar dataset functions when the algorithms run on the system for experiment purposes.

We ran all benchmark datasets, as defined in Table 5, on all existing task scheduling methods and the proposed method to evaluate the efficiency and effectiveness of all processes. We explain the detail of all datasets as follows. There are four columns in benchmarks datasets, such as the workload name, the data size of all tasks inside in the dataset, required CPU instruction (CIns) to run all assignments, and several tasks to be executed.

### 5.4. Component Calibration of Proposed Algorithm

The HCCETS has three components for calibration, such as task prioritizing, task scheduling and fuzzy based cost-efficient rescheduling. The study exploited RPD (relative percentage deviation) to evaluate the performance of the algorithm; the calculation of RPD is defined as follows:

(13) $$RPD\%=\frac{Z-Z^{*}}{Z^{*}}\times 100\%$$

Z is the initial task scheduling solution to the assigned task on the fog server j. On the contrary, $Z^{*}$ is the optimal solution among all solutions while any task has already been assigned to the fog server. 

### 5.5. Performance Metrics

There are many metrics to be taken into consideration for the experiment. Those are the error rate of tasks, deadlines, execution costs, bandwidth utilization costs, and QoS requirements of a task based on its deadline and cost constraints.

### 5.6. Baseline Approaches and System

This study compared the proposed system and algorithm with the following baseline approaches.

- Baseline 1: This study implement the heterogeneous earliest finish time [40] method to schedule autonomous healthcare application tasks to the heterogeneous clouds. This study processed all tasks through its different phases until completion.
- Baseline 2: This approach makes a topological order of processors such as fog cloud and by handing over their various priorities. This process is continuous in anticipation of a suitable schedule being gained [41].
- Base-Frame 1: This study implements existing healthcare [18] for IoMT applications that provide resources based on the heterogeneous cloud without any prioritizing tasks during scheduling.
- Base-Frame 2: This study implements the existing healthcare mobile cloud system [42], which offers services to the IoT application without a guaranteed deadline constraint.

### 5.7. Algorithm and System Comparison

**Figure 6:**
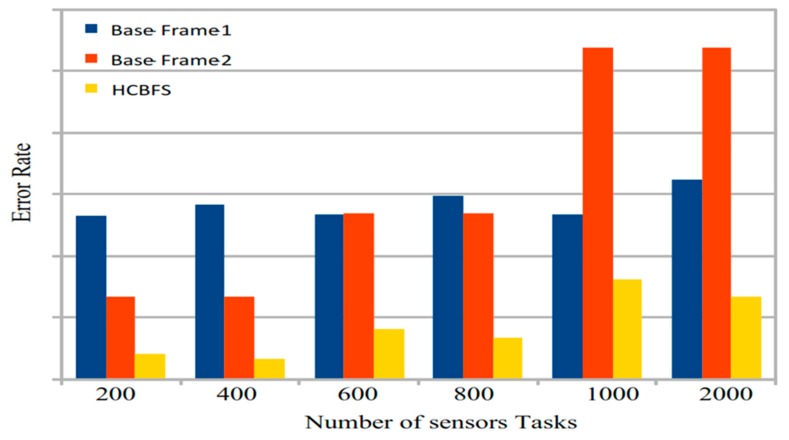
Error rate of task.

The healthcare based fog cloud system (HCBFS) is a cost-efficient system that ensures the task quality of experiment (QoE) of different tasks during assigning and processing in the heterogeneous fog server environment. The management of real-time generated data by different sensors and the stochastical arrival of tasks to the system is not easy. Therefore, the study estimated each task execution time, then prioritized them and performed initial scheduling without any delay. After that, the fuzzy-based efficient algorithm reschedules all tasks with minimum cost under their deadline requirements. Figure 6 illustrates that the HCBFS has a lower error rate (i.e., failure ratio of tasks) during offloading and scheduling on different heterogeneous fog servers. The current study did not focus on error ratea and the QoE of tasks, it only considered the scheduling situation without any deadline constraint. The study’s proposed framework adopts any environmental changes during the schedule, and reduces the applications and improves the overall performance as compared to the current static fog cloud architectures.

### 5.8. Task Scheduling

The proposed HCCETS is composed of different phases such as task prioritization, task scheduling, and a fuzzy-based cost-efficient rescheduling phase. It is similar to HEFT heuristics; however, HEFT did not directly apply to the cost-efficient task scheduling problem without any further improvement.

#### 5.8.1. Deadline Quality Aware Satisfaction

**Figure 7:**
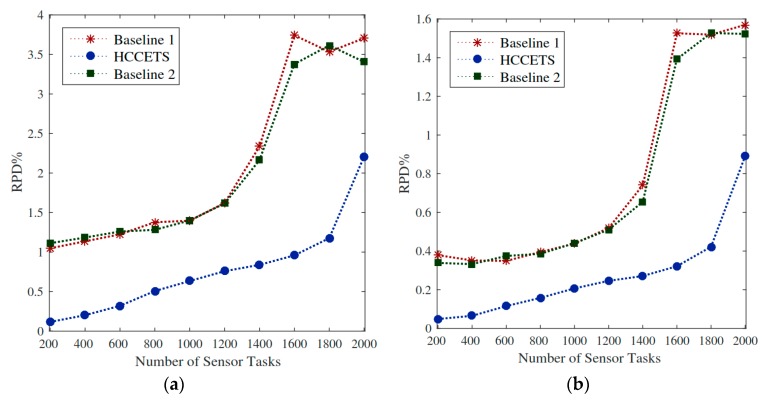
Objective function With deadline constraint. (**a**,**b**) the relative percentage ratio of the objective function.

**Figure 8:**
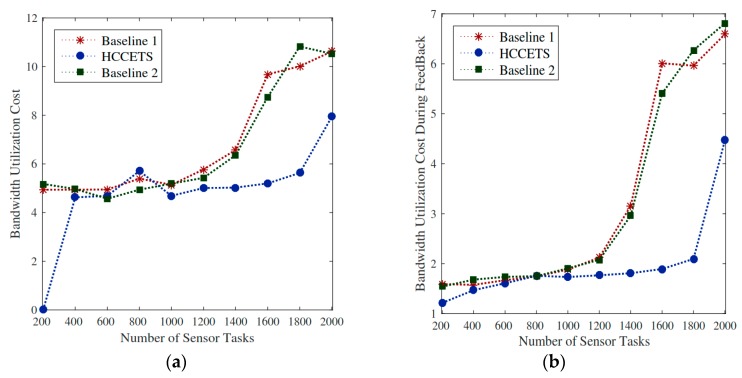
Utilization cost of fog servers. (**a**,**b**) shows that the relative percentage deviation of the HCCETS incurs lower utilization of metric bandwidth.

**Figure 9:**
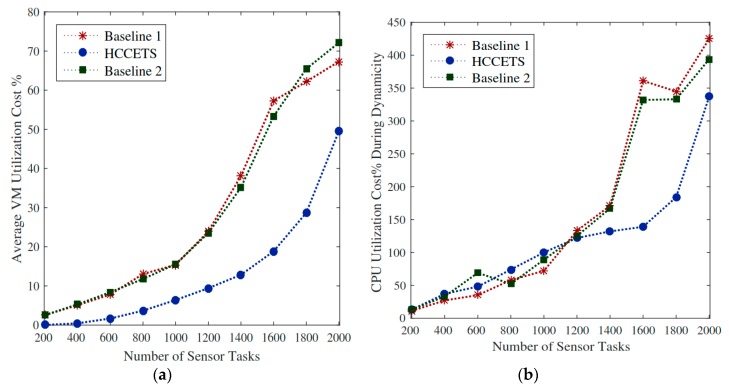
CPU utilization of fog servers. (**a**,**b**) the HCCETS incurs lower RPD% in terms of CPU utilization as compared to the existing Baseline 1 and Baseline 2.

**Figure 10:**
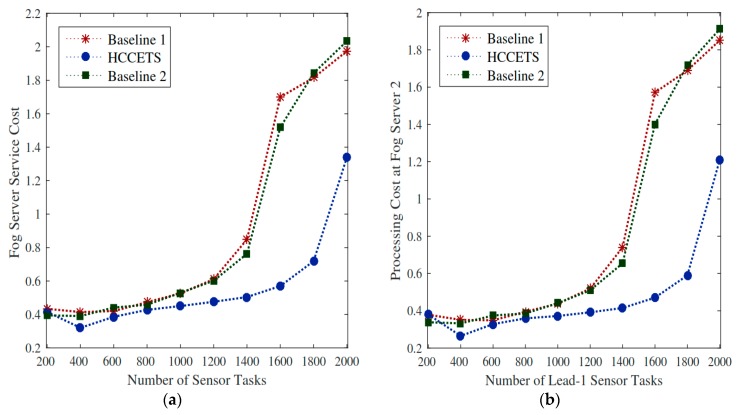
Scheduling of tasks to fog servers. (**a**,**b**) HCCETS improved system utilization cost as compared to the homogeneous system based Baseline 1 and Baseline 2.

**Figure 11:**
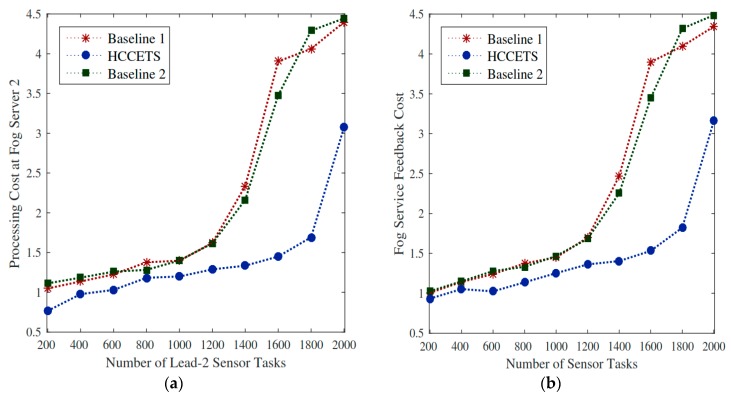
Rescheduling of tasks to fog servers. (**a**,**b**) the HCCETS incurs lower RPD% as compared to existing studies by rescheduling all pre-scheduled tasks to improve the efficacy of system utilization.

As the study suggests, the system schedules N numbers with deadlines into heterogeneous fog servers. It is significant to allocate critical healthcare tasks in a certain way that must execute in their deadlines. In this system, we have abandon computing resources to schedule requested under their deadlines. Generally, tasks miss their deadlines due to resource-constrained issues in the servers. Therefore, the author takes different fog servers with distinctive capacities to avoid any failure of job. This study sets the sum of deadlines for completing tasks under 2.5 points. Figure 7 shows the relative percentage ratio of the objective function while considering that the deadline metric lower while exploiting the proposed HCCETS framework. The main reason behind this is that Baseline 1 and baseline operated homogeneous fog cloud servers with limited resource capabilities often suffer from many failures of tasks during scheduling. Figure 7a,b proves that the RPD% of the objective is improved by exploiting HCCETS as compared to the existing baseline approaches. The main cause is that the existing baseline heuristics approach do not considered the rescheduling situations when they make task assignments to the heterogeneous clouds, whereas Figure 8, Figure 9, Figure 10 and Figure 11 show that HCCETS also reduces the cost of bandwidth utilization cost, CPU utilization cost, and task scheduling for all requested tasks.

#### 5.8.2. Bandwidth Utilization during Scheduling and Feedback Results

In the proposed HCBFS system, the placement distributed fog servers are very resilient in running IoT healthcare applications in an efficient manner. The bandwidth utilization of user devices when submitting tasks to the fog servers and getting back their feeds consume less bandwidth as compared to the existing scheduling methods. The principle behind that is that the scheduler chooses the nearest fog server for task execution to minimize bandwidth utilization cost. Figure 8a shows that the relative percentage deviation of the HCCETS while using bandwidth cost incurs lower utilization of metric bandwidth during scheduling as compared to the edge of computing existing methods. As Baseline 1 and Baseline 2 exploited sco-operative edge cloud and public clouds for bandwidth utilization, it requires a lot of bandwidth to send and receive tasks in the system. As it is similar to feedback result, Figure 8b illuminates HCCETS placed flexible and proximal to the user application and incurs lower feedback download cost as compared to the centric cloud.

#### 5.8.3. CPU Utilization Cost

IoMT applications consist of different kinds of tasks such as critical tasks and non-critical tasks. Risky jobs require immediate resources for execution. Generally, this process is costly and incurs extra cost during scheduling in the system. However, non-critical tasks may be scheduled into a tawdry fog cloud server because they have deadlines for their executions. The proposed HCCETS schedules critical functions to the high-performance fog servers (expensive cost), and non-critical tasks to the cheap fog servers; in this way, the author can maintain the overall cost of the applications. Figure 9a demonstrates that the HCCETS incurs lower RPD% in terms of CPU utilization as compared to the existing Baseline 1 and Baseline 2. Where existing studies exploited homogeneous fog cloud systems with steep costs, and scheduled all tasks on the same type of servers, it incurred unreasonable costs during scheduling. We considered the dynamic environment of network contents to the recognized problem, and it can be seen that Figure 9b HCCETS is an adaptive method during runtime changes in the system that doesn’t affect application performance.

#### 5.8.4. Initial Task Scheduling

Initially, this study scheduled all tasks based on available resources in the fog server environment under their deadline requirements. There is no wait time for each job in the system, because all tasks are scheduled immediately into heterogeneous fog clouds while satisfying their deadlines. As the author assumed to abandon resources regardless of servers, Figure 10a shows that HCCETS improved system utilization cost as compared to the homogeneous system based Baseline 1 and Baseline 2. The main limitation of the [40,41] homogenous system is that they have resource constraints and non-allocated tasks must wait until resources become free after some time. The swapping between high-cost fog server1 to low fog server 2 is quite useful once the scheduler does the initial schedule. HCCETS reshuffled tasks placement between fog servers to minimize the system cost, as shown in Figure 10b.

#### 5.8.5. Cost Efficient Rescheduling for All Tasks

This study rescheduled all tasks in a cost-efficient manner to improve the overall system costs as well as the bandwidth utilization cost of the IoMT applications. Similarly, existing Baseline 1 and Baseline 2 studies have only focused on the computational cost of servers regardless of the bandwidth utilization cost. Hence, Figure 11a proves that processing cost of fog server2 after swapping from fog server 1 incurs lower RPD% by the HCCETS as compared to existing studies. It is because rescheduling all pre-scheduled tasks from higher-cost fog server 1 to lower-cost fog server 2 reduces the system cost of applications. The placement of fog server 2 is flexible to users, and it gained lower feedback (e.g., download) results from price while exploited HCCETS framework. Figure 11b proved that HCCETS outperforms existing baseline approaches that did not focus the placement of their servers during the task scheduling problem.

## 6. Conclusions

In this paper, the author considered the cost-efficient task scheduling problem for healthcare-based heartbeat medical applications in fog cloud systems. The objective was to offer omnipresent cloud services to the generated data with minimum cost. For minimizing the total cost, the author proposed a novel health care based fog cloud system (HCBFS) which determines the processing of submitted tasks of the application. This study devised a health care awareness cost-efficient task scheduling (HCCETS) algorithm framework, which is not only schedules all tasks with minimum cost but executes them under their deadlines. Performance evaluation shows that the proposed task scheduling algorithm framework outperforms the existing algorithm methods in terms of cost. Fault tolerance is one of the major concerns to ensure the availability and reliability of services, as well as to perform the tasks. In order to minimize the impact of failure on the system and to ensure correct task execution, the system must be anticipated and be managed. Future work shall consider the fault tolerance, with security constraints on the fog cloud Internet of Thing medical applications. The HCCETS has some limitations, such as it does not support awareness mobility services, fault-tolerant cost, and energy cost of the system. The HCCETS does not focus on security costs. However, future work will focus on these aspects for further improvements.

## Figures and Tables

**Table 1:** Notations used for the problem.

Notation	Definition
*N*	The set of healthcare tasks *v*
*M*	Fog cloud networks *V*
$V_{j}$	The *j*th fog cloud
$\upsilon_{i}$	The *i*th healthcare task
$W_{i}$	The data of healthcare task $\upsilon_{i}$
$\zeta_{j}$	Computing rate of the fog server $V_{j}$
$p_{j}$	Cost of each fog cloud $V_{j}$
$T_{i}^{e}$	Calculated execution time $\upsilon_{i}$
$x_{i,j}$	Assignment of a task to a fog cloud
$Z_{i}$	It denotes the total execution cost of a task$\upsilon_{i}$
$F_{i}$	Completion of the task $\upsilon_{i}$
*TST*	Slack-timing of scheduling
$T_{i}^{s}$ lack	The lateness of a task $\upsilon_{i}$

**Table 2:** Simulation parameters.

Simulation Parameters	Values
Languages	Python, JAVA, CSharp
Simulation time	24 h
Experiment repetition	30 times
Program implementation	Eclipse
Lead I	DFR0027
Lead II	Arduino
N	2000
M	3

**Table 3:** Heterogeneous fog server resource specification.

Resource Type	Storage (GB)	Core	Speed (MIPS)	Cost-M
Fog Server 1	20,000	1	10,000	100 $
Fog Server 2	50,000	1	5000	200 $
Fog Server 3	100	1	1000	500 $

**Table 4:** The overall description of the heartbeat datasets.

Datasets	ECG Subjects	Patients	Critical Heartbeat	Non-Critical Heartbeat
MIT-BIH-SVDB	78	-	9953	174,317
MIT-BIH-ARR	48	47	7803	92,754
AHA	155	-	32,403	317,612

**Table 5:** Heartbeat datasets workload.

Workload	Data Size (MB)	C.Ins. (MI)	No. of Tasks
MIT-BIH-SVDM	500	5.8	825
MIT-BIH-AR	800	6.8	750
AHA	900	7.8	1000
